# Outcomes of non-vertex second twins, following vertex vaginal delivery of first twin: a secondary analysis of the WHO Global Survey on Maternal and Perinatal Health

**DOI:** 10.1186/1471-2393-14-55

**Published:** 2014-01-31

**Authors:** Joshua P Vogel, Erica Holloway, Cristina Cuesta, Guillermo Carroli, João Paulo Souza, Jon Barrett

**Affiliations:** 1 School of Population Health, Faculty of Medicine, Dentistry and Health Sciences, University of Western Australia, 35 Stirling Highway, Crawley, WA 6009, Australia; 2 UNDP/UNFPA/UNICEF/WHO/World Bank Special Programme of Research, Development and Research Training in Human Reproduction (HRP), Department of Reproductive Health and Research, World Health Organization, Avenue Appia 20, Geneva CH-1211, Switzerland; 3 Department of Obstetrics and Gynaecology, University of Toronto, Toronto, ON, Canada; 4 Centro Rosarino de Estudios Perinatales, Rosario, Santa Fe, Argentina

**Keywords:** Fetal presentation, Maternal and perinatal outcomes, Twin, Vertex

## Abstract

**Background:**

Mode of delivery remains a topic of debate in vertex/non-vertex twin pregnancies. We used the WHO Global Survey dataset to determine the risk of adverse maternal/perinatal outcomes associated with presentation of the second twin, following vaginal delivery of a vertex first twin.

**Methods:**

We analysed a derived dataset of twin pregnancies ≥ 32 weeks gestation where the first twin was vertex and delivered vaginally. Maternal, delivery and neonatal characteristics and adverse outcomes were reported by presentation of the second twin. Logistic regression models (adjusted for maternal and perinatal confounders, mode of delivery and region) were developed to determine odds of adverse outcomes associated with presentation.

**Results:**

1,424 twin pregnancies were included, 25.9% of these had a non-vertex second twin and Caesarean was more common in non-vertex presentations (6.2% vs 0.9%, p < 0.001). While the odds of Apgar < 7 at 5 minutes were higher in non-vertex presenting second twins (16.0% vs 11.4%, AOR 1.42 95% CI 1.01-2.00), the odds of maternal ICU admission (4.6% vs 1.7%, AOR 1.30, 95% CI 0.88-1.94), blood transfusion (6.0% vs 3.4%, AOR 1.23, 95% CI 0.67-2.25), stillbirth (7.6% vs 4.7%, AOR 1.15, 95% CI 0.72-1.73), early neonatal death (3.8% vs 2.1%, AOR 1.68, 95% CI 0.96-2.94), and NICU admission (26.6% vs 23.2%, AOR 0.93, 95% CI 0.62-1.39) were not.

**Conclusion:**

After a vaginal delivery of a vertex first twin, non-vertex presentation of the second twin is associated with increased odds of Apgar <7 at 5 minutes, but not of other maternal/perinatal outcomes. Presentation of the second twin is not as important a consideration in planning twin vaginal birth as previously considered.

## Background

The mode of delivery of the second twin remains a topic of debate in modern obstetrics, particularly when the first twin is vertex and the second twin is in breech presentation. Caregivers often extrapolate the findings of the Term Breech Trial that demonstrated a significant reduction in adverse perinatal outcome with planned caesarean delivery of the term breech singleton [1], however most practitioners realize the limitations of this extrapolation. While evidence suggests that the second twin is at increased risk of perinatal morbidity at all gestational ages, [2] some experienced practitioners have expressed the opinion that the second twin presenting breech is at less risk than the second twin presenting vertex, as it may be delivered by breech extraction [3].

The second twin presents non-vertex in approximately 40% of twin gestations prior to the onset of labour [4] and fetal presentation and mode of delivery of the second twin are known determinants of adverse neonatal outcomes [2,5,6]. Intrapartum complications that place the second twin at risk following vaginal delivery of the first twin include placental abruption, intrapartum haemorrhage, cord prolapse, difficulty in monitoring the fetal heart rate and fetal bradycardia [7].

Two large population-based retrospective cohort studies suggest that elective caesarean delivery of twins may improve perinatal outcome in the second twin [5,8]. A single randomised control trial (RCT) of 60 pairs of twins (second twin presenting non-vertex) compared caesarean to vaginal delivery. This trial demonstrated a significant increase in maternal febrile morbidity and a trend towards increased use of general anaesthetic in patients undergoing caesarean delivery. No perinatal deaths or significant neonatal morbidities were identified in either study group [9]. This RCT was included in a Cochrane review that concluded there was inadequate evidence to recommend routine caesarean delivery in twin gestations with non-vertex presentation of the second twin – given the small sample size of the study with limited power [10]. A large multicenter RCT, the Twin Birth Study has recently concluded. This study has shown that planned vaginal birth is as safe as planned lower segment caesarean section (LSCS) for twins between 32 and 38 weeks gestation [11]. In addition, subgroup analysis did not show any effect on the presentation of the second twin on the primary outcome. However, even in this large (n = 2,795 fetuses) trial, subgroup analysis will be prone to random error.

This study aimed to determine whether presentation of the second twin following vaginal delivery of a vertex first twin was associated with poorer maternal or neonatal outcomes, after adjusting for mode of delivery. In addition, we aimed to address the paucity of data on this issue from low- and middle-income settings, where the rates of adverse maternal and perinatal outcomes are higher [12] and, in many African countries, twin pregnancies are more frequent [13]. To this end, we conducted a secondary analysis of twin deliveries in the WHO Global Survey on Maternal and Perinatal Health (WHOGS), a large, multi-centre, cross-sectional survey of deliveries in 24 countries.

## Methods

### Study design, setting and participants

The WHO Global Survey on Maternal and Perinatal Health (WHOGS) was a multi-country, multicentre survey designed to collect information regarding mode of delivery and its impact on maternal and perinatal health outcomes. Methodological details of the WHOGS have been published elsewhere [14,15]. A stratified multistage cluster sampling design was used to obtain a random sample of institutions from countries and health institutions worldwide. Countries in the WHO regions were grouped according to adult and under-five infant mortality. From each of these sub regions, four countries were randomly selected, with probability proportional to population size. The study was implemented in 24 countries in Africa, Latin America and Asia. In each country, the capital city was selected, along with two randomly selected provinces (probability proportional to population size). From within these, a census of all facilities with more than 1,000 births per year and capacity to perform caesarean sections was obtained. If there were more than seven facilities, seven were randomly selected (probability of selection proportional to the number of births per year). If there were fewer than seven facilities, all were selected. Participating facilities captured data on all deliveries occurring over a three month period. The WHOGS captured 290,610 deliveries and was conducted over 2004 and 2005 (Africa and Latin America) and 2007 and 2008 (Asia). Individual-level data on women and their babies was abstracted by trained data collectors from the medical record; there was no contact between data collectors and patients. After collection, data were entered at the country, provincial or facility level in a web-based system (MedSciNet AB, Stockholm, Sweden).

### Variables, confounders and outcomes

The WHOGS individual dataset includes demographic characteristics, obstetric and medical history, mode of delivery and maternal and perinatal outcomes up to discharge from hospital, day 7 postpartum or death, whichever occurred first. Morbidity and mortality occurring post-discharge, or during a subsequent readmission were not captured. Maternal medical and obstetric conditions (such as cardiac/renal disease or pre-eclampsia) were recorded as binary variables (yes/no); severity, time of onset and management were not captured. Continuous variables (maternal age, education, parity, gestational age and birthweight) were converted to categorical variables for analysis. Chorionicity was not captured in the WHOGS, however we reported the prevalence of sex discordant twin pregnancies.

We developed an *a priori* list of confounders based on variables available in the WHOGS and clinical and epidemiological evidence in the literature. However, due to low numbers of cases, some were collapsed into a composite variable. The maternal-level confounders included were: maternal age (<20, 20-35, >35), maternal education (0, 1-4, 5-9, > = 10), parity (0, 1-2, > = 3), antenatal visits (0, 1-3, > = 4), mode of delivery (vaginal or caesarean), hypertensive diseases (chronic or pregnancy-induced hypertension), malaria, other medical diseases (HIV, pregestational diabetes, cardiac/renal disease, chronic respiratory conditions, sickle cell anaemia), prelabour rupture of membranes, pre-eclampsia/eclampsia, vaginal bleeding in 2^nd^ half of pregnancy and urine infection/pyelonephritis. Perinatal-level confounders were sex and sex discordance (yes/no), gestational age (<37 or > =37 weeks), birthweight (<2500 g or > =2500 g) and birthweight discordance (<15% discordance, larger or smaller twin of a >15% birthweight discordant pair). The exposure variable of interest was presentation of the second twin, i.e. group 1 (non-vertex presentation) and group 2 (vertex presentation). The maternal outcomes were maternal death, ICU admission, blood transfusion, hysterectomy or 3^rd^/4^th^ degree perineal laceration within the seven days following birth. The perinatal outcomes were stillbirth (newborn with no signs of life), early neonatal mortality in a liveborn neonate up to hospital discharge or seven days of life, Apgar score <7 at 5 minutes and admission of the newborn to NICU.

### Analysis and statistical methods

For this analysis, a specific database derived from the WHOGS database was created including twin pregnancies only as the unit of analysis. Twin pregnancies were included if a) the first twin delivered vaginally in vertex presentation and b) the gestational age was greater than or equal to 32 weeks. These women were then categorized into groups 1 and 2, based on the presentation of the second twin at the time of delivery. We described the regional and country distribution of twin pregnancies and reported frequencies for maternal demographic characteristics, obstetric history, mode of delivery and neonatal characteristics by presentation of the second twin. All maternal and neonatal outcomes were reported similarly. Chi-square tests, adjusted for survey design (using Complex Samples module in SPSS 20, with strata = country and cluster = facility) were used to test significance; p-values of less than 0.05 were considered significant.

Risks of maternal and perinatal outcomes associated with presentation of the second twin were determined using generalized linear mixed models (GENLINMIXED) with facility and country as random effects, to account for clustering of individuals within facilities and facilities within countries. Models of maternal outcomes were adjusted for maternal-level confounders only, whereas perinatal models were adjusted for maternal- and perinatal-level confounders. Both crude and adjusted odd ratios with corresponding 95% confidence intervals (95% CI) were reported and all missing values were excluded from all modeling. All statistical analysis was conducted using SPSS 20 [16]. Ethical clearance from all Ministries of Health of participating countries, WHO Ethics Review Committee and sub-regional ethical boards was obtained.

## Results

**Figure 1:**
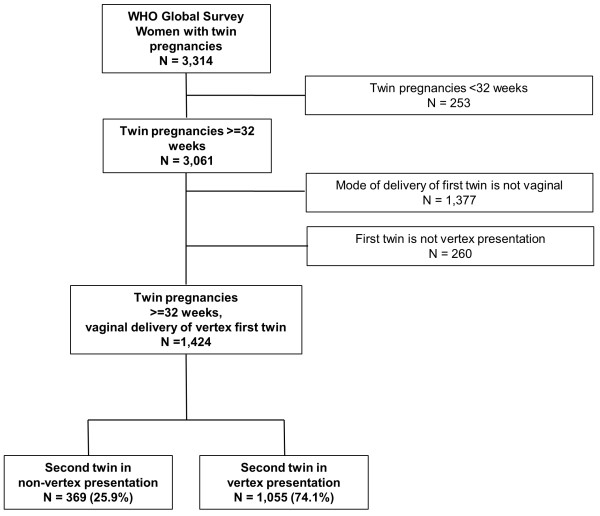
Study profile.

The WHOGS database included 3,314 twin pregnancies. This analysis included the 1,424 twin pregnancies > = 32 weeks gestation in which the first twin delivered vaginally in vertex presentation. The second twin was non-vertex in 25.9% of twin pregnancies (Figure 1). 53% of these were from African countries, 33.0% from Asian countries and 14.0% from Latin American countries (Table 1). The two study groups were similar with respect to maternal age, marital status, maternal education, parity, history of caesarean section at last pregnancy, antenatal care and onset of labour (Table 2). Delivery by caesarean section was significantly more common in non-vertex than vertex presentations (6.2% vs 0.9%, p < 0.001). Rates of infant gender, preterm delivery, low birthweight and birthweight discordance were similar between groups. In the 23 non-vertex second twins delivered by caesarean, the most frequent documented indications were non-vertex presentation (n = 13, 59.1%) and fetal distress (n = 5, 22.7%). In the 10 vertex second twins delivered by caesarean, the most frequent indication was multiple pregnancy (n = 7, 70%) and cephalo-pelvic disproportion (n = 3, 30%). The included twin pregnancies occurred in 265 facilities, of which most were urban (75.5%), secondary (47.2%) or tertiary (39.2%) facilities, and had neonatal intensive care (53.2%) and ultrasound (80.8%) in the facility (Table 3).

**Table 1:** Number of twin pregnancies by region and country

**Country/Region**	N (%*)
**Africa**	**755 (53.0)**
Algeria	146 (10.3)
Angola	46 (3.2)
Democratic Republic of Congo	98 (6.9)
Kenya	150 (10.5)
Niger	103 (7.2)
Nigeria	119 (8.4)
Uganda	93 (6.5)
**Asia**	**470 (33.0)**
Cambodia	50 (3.5)
China	23 (1.6)
India	171 (12.0)
Japan	9 (0.6)
Nepal	40 (2.8)
Philippines	64 (4.5)
Sri Lanka	60 (4.2)
Thailand	23 (1.6)
Vietnam	30 (2.1)
**Latin America**	**199 (14.0)**
Argentina	27 (1.9)
Brazil	30 (2.1)
Cuba	35 (2.5)
Ecuador	15 (1.1)
Mexico	27 (1.9)
Nicaragua	6 (0.4)
Paraguay	7 (0.5)
Peru	52 (3.7)
**TOTAL**	**1,424 (100)**

*% refers to the percentage of overall total of twin pregnancies (twin pregnancies [country])/all twin pregnancies.

**Table 2:** Characteristics of mothers and newborns, by fetal presentation of the second twin

	**Non-vertex n/N, (%) N = 369**	**Vertex n/N, (%) 1,055**	**Adjusted chi square P value**^**a**^
**Age (years)**			
<20	23/369 (6.2)	65/1053 (6.2)	0.247
20-35	305/369 (82.7)	901/1053 (85.6)	
>35	41/369 (11.1)	87/1053 (8.3)	
**Marital status**			
Married	337/367 (91.8)	967/1053 (91.8)	0.997
Not married	30/367 (8.2)	86/1053 (8.2)	
**Maternal education (years)**			
Nil	63/348 (18.1)	139/999 (13.9)	0.244
1 – 4	17/348 (4.9)	64/999 (6.4)	
5 – 9	140/348 (40.2)	430/999 (43.0)	
> = 10	128/348 (36.8)	366/999 (36.6)	
**Parity**			
Nil	103/368 (28.0)	270/1052 (25.7)	0.559
1 or 2	164/368 (44.6)	498/1052 (47.3)	
> = 3	101/368 (27.4)	284/1052 (27.0)	
**Caesarean delivery at last pregnancy**			
Yes	7/329 (2.1)	14/941 (1.5)	0.434
No	322/329 (97.7)	927/941 (98.5)	
**Antenatal care**			
Nil	25/344 (7.3)	76/983 (7.7)	0.850
1 to 3	107/344 (31.1)	319/983 (32.5)	
> = 4	212/344 (61.6)	588/983 (59.8)	
**Labour**			
Spontaneous	341/369 (92.4)	961/1055 (91.1)	0.691
Induced	25/369 (6.8)	87/1055 (8.2)	
No labour	3/369 (0.8)	7/1055 (0.7)	
**Mode of delivery**			
Vaginal delivery	346/369 (93.8)	1045/1055 (99.1)	<0.001
Caesarean section	23/369 (6.2)	10/1055 (0.9)	
**Birth attendance at delivery**			
Doctor	163/369 (44.2)	427/1055 (40.5)	0.054
Midwife/Nurse	198/369 (53.7)	568/1055 (53.8)	
Other	8/369 (2.2)	60/1055 (5.7)	
**Infant sex**			
Male	190/369 (51.5)	530/1055 (50.2)	0.704
Female	179/369 (48.5)	525 (49.8)	
**Sex discordance**			
Twins are same gender	234/369 (63.4)	779/1055 (73.8)	<0.001
Twins are different gender	135/369 (36.6)	276/1055 (26.2)	
**Congenital malformation**			
Yes	3/369 (0.8)	6/1055 (0.6)	0.628
No	366/369 (99.2)	1049/1055 (99.4)	
**Gestational age at delivery**			
<37 weeks	117/369 (31.7)	288/1055 (27.3)	0.110
> = 37 weeks	252/369 (68.3)	767/1055 (72.7)	
**Birthweight at delivery**			
<2500 g	222/367 (60.5)	570/1054 (54.1)	0.067
> = 2500 g	145/367 (39.5)	484/1054 (45.9)	
**Birthweight discordance**			
Birthweight discordance <15%	243/369 (65.9)	753/1055 (71.4)	0.123
Larger twin of a >15% birthweight discordant pair	51/369 (13.8)	132/1055 (12.5)	
Smaller twin of a >15% birthweight discordant pair	75/369 (20.3)	170/1055 (16.1)	

^a^Chi-square p values are adjusted for survey design; <0.05 considered significant.

**Table 3:** Characteristics of facilities

	**Facility characteristics**	
	**N = 265**	
	**n**	**%**
**Location**		
Urban	200/265	75.5
Peri-urban	27/265	10.2
Rural	36/265	13.6
Missing	2/265	0.8
**Level of facility**		
Primary	0/265	0.0
Secondary	125/265	47.2
Tertiary	104/265	39.2
Other referral level	30/265	11.3
Missing	6/265	2.3
**Availability of neonatal intensive care**		
Yes	141/265	53.2
No	124/265	46.8
Missing	0/265	0
**Availability of ultrasound services**		
Yes	214/265	80.8
No	51/265	19.2
Missing	0/265	0

Adjusted odds ratios for the outcomes maternal death, hysterectomy and 3^rd^/4^th^ degree perineal lacerations could not be calculated due to low numbers. Perineal laceration was not significantly higher among vertex presentations (1.4% vs 1.9%, OR 0.71, 95% CI 0.27 – 1.91) (Table 4). The adjusted odds of maternal ICU admission (AOR 1.30, 95% CI 0.88 – 1.94) and blood transfusion (AOR 1.23, 95% CI 0.67 – 2.25) were not significantly higher in non-vertex presentations. The one maternal death reported in this study population followed a vertex/vertex vaginal delivery. There was a small significant increase in the odds of Apgar score <7 at 5 minutes (AOR 1.42, 95% CI 1.01 – 2.00), but stillbirth (AOR 1.15, 95% CI 0.72 – 1.73), early neonatal mortality (AOR 1.68, 95% CI 0.96 – 2.94) and admission to NICU (AOR 0.93, 95% CI 0.62 – 1.39) were not (Table 5).

**Table 4:** Maternal outcomes, by fetal presentation of the second twin

	**Non-vertex n/N, (%) N = 369**	**Vertex n/N, (%) 1,055**	**Crude odds ratio (95% CI)**	**Adjusted odds ratio**^**a **^**(95% CI)**
Maternal death	0/369 (0.0)	1/1055 (0.1)	*	*
ICU admission	17/369 (4.6)	18/1055 (1.7)	2.78 (1.42 – 5.45)	1.30 (0.88 – 1.94)
Blood transfusion	22/369 (6.0)	35/1055 (3.4)	1.84 (1.07 – 3.18)	1.23 (0.67 – 2.25)
Hysterectomy	0 / 369 (0.0)	0/1055 (0.0)	*	*
3^rd^/4^th^ degree perineal laceration	5/369 (1.4)	20/1055 (1.9)	0.71 (0.27 – 1.91)	*

*Cannot calculate odds ratios as too few cases.

^a^Generalized linear mixed models adjusted for: maternal age (<20, 20-35, >35), maternal education (0, 1-4, 5-9, > = 10), parity, (0, 1-2, > = 3), antenatal visits (0, 1-3, > = 4), mode of delivery (vaginal or caesarean), hypertensive diseases (chronic or pregnancy-induced hypertension), malaria, other medical diseases (HIV, pregestational diabetes, cardiac/renal disease, chronic respiratory conditions, sickle cell anaemia), prelabour rupture of membranes, pre-eclampsia/eclampsia, vaginal bleeding in 2^nd^ half of pregnancy and urine infection/pyelonephritis; facility and country as random effects.

**Table 5:** Perinatal outcomes, by fetal presentation of the second twin

	**Non-vertex n/N, (%) N = 369**	**Vertex n/N, (%) 1,055**	**Crude odds ratio (95% CI)**	**Adjusted odds ratio**^**a **^**(95% CI)**
Stillbirth	28/369 (7.6)	50/1055 (4.7)	1.65 (1.02 – 2.66)	1.15 (0.72 – 1.73)
Early neonatal mortality	14/369 (3.8)	22/1055 (2.1)	1.86 (0.94 – 3.67)	1.68 (0.96 – 2.94)
Apgar score <7 at 5 minutes	59/369 (16.0)	120/1050 (11.4)	1.48 (1.05 – 2.07)	1.42 (1.01 – 2.00)*
Admission to NICU	98/369 (26.6)	245/1055 (23.2)	1.20 (0.91 – 1.58)	0.93 (0.62 – 1.39)

^a^Generalized linear mixed models adjusted for: maternal age (<20, 20-35, >35), maternal education (0, 1-4, 5-9, > = 10), parity, (0, 1-2, > = 3), antenatal visits (0, 1-3, > = 4), mode of delivery (vaginal or caesarean), hypertensive diseases (chronic or pregnancy-induced hypertension), malaria, other medical diseases (HIV, pregestational diabetes, cardiac/renal disease, chronic respiratory conditions, sickle cell anaemia), prelabour rupture of membranes, pre-eclampsia/eclampsia, vaginal bleeding in 2^nd^ half of pregnancy and urine infection/pyelonephritis, sex, sex discordance (yes/no), gestational age (<37 or > =37 weeks), birthweight (<2500 g or > =2500 g) and birthweight discordance (<15%, larger twin of >15% birthweight discordant pair, smaller twin of >15% birthweight discordant pair); country and facility as random effects.

## Discussion

We conducted a logistic regression analysis of a multi-country, facility-based survey dataset of twin pregnancies and determined that, following vaginal delivery of the vertex first twin, there was a significant increase in the odds of Apgar <7 at 5 minutes in non-vertex presenting second twins. However, the odds of maternal ICU admission, blood transfusion, stillbirth, early neonatal mortality or admission to NICU were not increased significantly. The absence of significantly increased odds of stillbirth or early neonatal mortality suggests that the presentation of the second twin is not as an important a prognostic feature as was previously thought.

To evaluate maternal and perinatal outcomes following delivery of these twin gestations, we analysed data from primarily urban, relatively large centres through this international survey. While the compared groups were similar in terms of maternal and perinatal characteristics, the higher rate of caesarean section in non-vertex presentations is suggestive of a preponderance for combined deliveries in this scenario, although the rate of caesarean in the vertex-presenting second twins in our study (0.9%) is considerably lower than that reported by Yang et al (6.3%) [7] and Wen et al. (9.5%) [17] in United States population-based studies; we believe this reflects the lower rate of caesarean in the WHOGS participating countries [18].

There were too few cases of maternal deaths, hysterectomies and perineal lacerations for regression analysis. The odds of maternal admission to ICU and blood transfusion were not higher in non-vertex presentations, however it is worthy of note that these two outcomes are proxy indicators of severe maternal morbidity and risk associations may be diluted due to limited access or unaffordability of these services in resource-constrained settings. In addition, the sampling frame for the WHOGS was primarily larger, urban facilities with caesarean section capacity. As higher risk or more complex pregnancies (such as twin pregnancies) are referred to these facilities, the rate of maternal morbidity in these facilities is likely higher than average, copared to the population or lower-order facilities.

In the WHOGS dataset, we have previously shown that twins had higher stillbirth (4.0%) and early neonatal death (3.1%) rates than singletons (1.7% and 0.6%) [19]. The stillbirth rate (7.6% and 4.7%) and the early neonatal mortality rate (3.8% and 2.1%) for both groups was substantially greater than in similar studies, [20-22] which we attributed to the higher underlying perinatal mortality in these countries, related to both the general health of the population and the availability of health care and health care providers. Also, Minakami and Sato demonstrated that the risk of fetal death in twins is significantly higher at and beyond 38 weeks [23] and 539 (37.9%) twin pregnancies included in this analysis were >38 weeks. While the presence of moderate to severe growth discordance in twin gestations is associated with increased perinatal morbidity and mortality, [24] rates of birthweight discordance were not significantly different between groups and regression models were adjusted for this important confounder. The odds of Apgar <7 at 5 minutes were higher in non-vertex presentations but stillbirth and early neonatal death were not; this supports the results of a meta-analysis of second twins by Rossi et al. which found that mortality rates between non-vertex and vertex second twins were similar (1.7% vs 1.1%, p = 0.60) [25]. The Twin Birth Study has shown that planned vaginal birth is not associated with an increase in adverse outcome for twin deliveries, compared to planned CS [11]. In addition, that study also showed that the presentation of the second twin after delivery of the first twin did not influence the primary outcome (a composite of perinatal mortality and morbidity).

This analysis has several strengths. It is based on a large, multi-country survey that captured a large number of twin pregnancies in 24 countries and is the largest study of this type from primarily low- and middle-income settings where perinatal mortality is often considerably higher and includes many African countries where twinning is more common than in other countries [13]. We had comprehensive information on maternal medical and obstetric history, allowing adjustment for multiple confounders. Most observational studies in this area have been conducted in higher-resource settings where rates of maternal and perinatal mortality and morbidity are lower, [25] making it more difficult to detect changes in outcomes. In addition, most twin studies have focused on perinatal morbidity and mortality without considering adverse maternal outcomes [17,25,26]. However, this analysis is not without its limitations. This study was not randomised and fetal presentation may have affected the selection of mode of delivery. As the WHOGS is a facility-based survey of primarily urban facilities, there are likely higher rates of morbidity and mortality referred to these facilities than in communities or lower-order facilities. Additionally, low- and middle-income countries participating in the WHOGS generally have higher rates of perinatal mortality, as well as significant at-risk sub-populations (such as adolescent and poorly educated mothers) compared to higher-income countries. Thus, these findings can only be extrapolated to similar contexts. Furthermore, it is possible that a larger study of twin pregnancies may be able to demonstrate significance in those outcomes that were found to not be significant in our study. While data collection procedures were standardised across facilities for the WHOGS, facilities and countries may differ in their protocols for management of twin pregnancies. Adjusting the logistic regression models for facility and country as random effects can only partially mitigate this heterogeneity. Suboptimal medical documentation may have affected data quality as the WHOGS utilised retrospective medical record review for data collection. While the magnitude of this is difficult to estimate, the primary maternal and perinatal outcomes of the WHOGS were the same as this analysis and missing rates were generally low, except for history of previous caesarean section at last pregnancy (10.8% missing). Other relevant variables were not captured by the WHOGS, such as time between twin deliveries, length and difficulty of labour, labour augmentation practices, indications for ICU admission and newborn laboratory parameters. While monochorionic twins are at increased risk for adverse perinatal outcome in comparison to dichorionic twin gestations, [27] chorionicity was not captured in the WHOGS and we were unable to compare outcomes between monochorionic and dichorionic twins.

## Conclusion

This analysis was designed to evaluate the association between fetal presentation of the second twin (following vaginal delivery of the vertex first twin) and short-term maternal and neonatal outcomes. There was no significant increase in maternal and perinatal outcomes associated with non-vertex presentation of the second twin, aside from a small increase in the odds of Apgar score <7 at 5 minutes for non-vertex presenting second twins. Importantly, mortality and NICU admission were not significantly higher with a non-vertex presentation. This is consistent with the findings of the large RCT, suggesting that the presentation of the second twin is not as an important consideration in planning twin vaginal birth as previously considered.

## Competing interests

The authors declare that they have no competing interests.

## Authors’ contributions

All the named authors helped conceive of the analysis and participated in its design. CC and JPV conducted the analysis. JPV drafted the manuscript and JPS and JB helped draft the manuscript. All authors read and approved the final manuscript. The views contained herein are the views of the named authors only.

## Pre-publication history

The pre-publication history for this paper can be accessed here:

http://www.biomedcentral.com/1471-2393/14/55/prepub
